# Contextual Factors That Impact the Implementation of Patient Portals With a Focus on Older People in Acute Care Hospitals: Scoping Review

**DOI:** 10.2196/31812

**Published:** 2023-02-03

**Authors:** Zarnie Khadjesari, Julie Houghton, Tracey J Brown, Helena Jopling, Fiona Stevenson, Jennifer Lynch

**Affiliations:** 1 Behavioural and Implementation Science research group School of Health Sciences University of East Anglia Norwich United Kingdom; 2 Norwich Medical School University of East Anglia Norwich United Kingdom; 3 Department of Public Health West Suffolk NHS Foundation Trust Bury St Edmunds United Kingdom; 4 Department of Primary Care and Population Health University College London London United Kingdom; 5 School of Health and Social Work University of Hertfordshire Hatfield United Kingdom

**Keywords:** patient portal, tethered personal health records, acute care hospitals, implementation, scoping review

## Abstract

**Background:**

Older people are the highest users of health services but are less likely to use a patient portal than younger people.

**Objective:**

This scoping review aimed to identify and synthesize the literature on contextual factors that impact the implementation of patient portals in acute care hospitals and among older people.

**Methods:**

A scoping review was conducted according to the PRISMA-ScR (Preferred Reporting Items for Systematic Reviews and Meta-Analyses Extension for Scoping Reviews) guidelines. The following databases were searched from 2010 to June 2020: MEDLINE and Embase via the Ovid platform, CINAHL and PsycINFO via the EBSCO platform, and the Cochrane Library. Eligible reviews were published in English; focused on the implementation of tethered patient portals; included patients, health care professionals, managers, and budget holders; and aimed at identifying the contextual factors (ie, barriers and facilitators) that impact the implementation of patient portals. Review titles and abstracts and full-text publications were screened in duplicate. The study characteristics were charted by one author and checked for accuracy by a second author. The NASSS (Non-adoption, Abandonment, Scale-up, Spread, and Sustainability) framework was used to synthesize the findings.

**Results:**

In total, 10 systematic reviews published between 2015 and 2020 were included in the study. Of these, 3 (30%) reviews addressed patient portals in acute care hospitals, and 2 (20%) reviews addressed the implementation of patient portals among older people in multiple settings (including acute care hospitals). To maximize the inclusion of the literature on patient portal implementation, we also included 5 reviews of systematic reviews that examined patient portals in multiple care settings (including acute care hospitals). Contextual factors influencing patient portal implementation tended to cluster in specific NASSS domains, namely the condition, technology, and value proposition. Certain aspects within these domains received more coverage than others, such as sociocultural factors and comorbidities, the usability and functionality aspects of the technology, and the demand-side value. There are gaps in the literature pertinent to the consideration of the provision of patient portals for older people in acute care hospitals, including the lack of consideration of the diversity of older adults and their needs, the question of interoperability between systems (likely to be important where care involves multiple services), the involvement of lay caregivers, and looking beyond short-term implementation to ways in which portal use can be sustained.

**Conclusions:**

We identified important contextual factors that impact patient portal implementation and key gaps in the literature. Future research should focus on evaluating strategies that address disparities in use and promote engagement with patient portals among older people in acute care settings.

## Introduction

### Background

Patient portals (also known as tethered personal health records) consist of an internet-based application that accesses the electronic health record of a health care organization and provides timely access to medical records, laboratory results, appointment bookings, repeat prescriptions, and secure messaging with health care professionals, among other content and functionality [1]. Patient portals aim to engage patients and carers in managing their care, which has been found to improve health outcomes, the quality of care, and patient safety [2]. Patient portals are well established in UK family practice, with electronic health records being commonplace in 96% of general practices for almost 3 decades [3]. In UK acute care hospitals, the use of handwritten inpatient records remains widespread [3], and as such, patient portals are less common. Global Digital Exemplar (GDE) trusts are internationally recognized providers of exceptional and efficient National Health Service (NHS) care via world-class digital technology and information and are committed to sharing best practices and supporting the widespread adoption of patient portals [4]. The future vision of the NHS is to create a single access point to acute care hospitals with integrated systems that share and exchange data securely with other health and care providers [5]. However, the integration of portals with the existing systems is currently a barrier to their adoption, in addition to clinical engagement, information governance, low patient awareness, and resources [6]. Furthermore, among the patients who currently access portals, engagement or meaningful use is often limited.

The greatest benefit to patients and the health service can be achieved by optimizing portal use among older people [7]. Older people (aged ≥65 years) are less likely to use a patient portal than younger people (86% of adopters are aged <65 years) [8], yet they are the highest users of the health service, with more than half (54%) of them experiencing multimorbidity [9]. Older people are more at risk of serious complications and hospital-acquired infections, and they may experience frailty and other mobility problems that hinder their access to health centers. Moreover, older age is the greatest risk factor for mortality from COVID-19 [10]. Barriers are exacerbated when older patients lack access to and experience of using technology, have lower levels of education, and have low health literacy [11-13]. However, older people have been found to express interest in using a patient portal independently or with a carer, irrespective of their health literacy level, previous portal use, or experience seeking health information over the internet [11]. The COVID-19 pandemic has accelerated the rate of adoption of digital technologies in health care settings by necessitating remote visits, communication, and monitoring, which are especially important for people managing long-term health conditions [14]. The need and demand for more flexible access to health services are unlikely to diminish.

There are numerous reviews of patient portal features, functionality, adoption, and implementation, with the vast majority focusing on family practice settings. A review examining portal use in multiple health care settings was published by Antonio et al [14]. This umbrella review explored the current state of evidence for patient portals, with a specific focus on portal technology. It identified several factors that influence portal adoption, including patient circumstances, interest, and satisfaction; portal usability; provider attitudes; and service use [14]. Another review across multiple health care settings reported a range of patient characteristics that impact portal use, such as age, ethnicity, education, health literacy, health status, and carer role [7], and factors that impact patient portal engagement, such as provider endorsement and portal usability. The authors argue that future research should aim to boost portal engagement among specific populations most likely to benefit from its use [7]. This review set out to scope the literature on older people and acute care settings to inform the broader aim of our research program—to develop an evidence-based implementation strategy for portal use and engagement among older people in acute care settings. Despite efforts toward the widespread uptake of and engagement with patient portals across acute NHS trusts, there is limited research into evidence-based strategies for successful engagement [15]. Engagement strategies, such as advertisement campaigns or training for patients, carers, and staff, must be tailored to the targeted population groups and the local context to be effective [16]. Tailoring can improve equity within the patient group. Identifying contextual factors that impact the routine implementation of patient portals in acute care hospitals is the first step toward developing an evidence-based implementation strategy for older people.

### Aim

The aim of this scoping review was to identify and synthesize the literature on contextual factors that impact the implementation of patient portals in acute care hospitals and among older people.

Our primary interest was to improve the engagement with patient portals among older people in acute care hospitals. However, we needed a broad scope of the literature to capture learning from studies in multiple settings (family practice and acute care hospitals) and patient groups (general population and older people), given the potential generalizability of the findings across settings.

## Methods

### Reporting Guidance and Theoretical Framework

This scoping review followed the methodological framework described by Arksey and O’Malley [17] and the PRISMA-ScR (Preferred Reporting Items for Systematic Reviews and Meta-Analyses Extension for Scoping Reviews) reporting guidelines [18]. The framework was selected to achieve our broad aim of summarizing what is known about our primary area of interest, to synthesize findings, and to highlight key gaps in the literature.

### Non-adoption, Abandonment, Scale-up, Spread, and Sustainability Framework

To build on the existing literature, we used a theoretical framework to provide a semantic structure to the synthesis of our findings. The NASSS (Non-adoption, Abandonment, Scale-up, Spread, and Sustainability) framework [19] provided the basis for summarizing the results. This framework was developed to analyze the varied outcomes of technological innovations in health and social care and to help inform the implementation of such technologies. The NASSS framework comprises 7 domains: (1) the condition or illness, (2) the technology, (3) the value proposition, (4) the adopter system (intended users), (5) the organization, (6) the wider system (especially regulatory, legal, and policy issues), and (7) a final cross-cutting domain that considers adaptation and embedding over time. Each of the 7 domains can be “simple” (ie, few components and predictable), “complicated” (ie, many components but still largely predictable), or “complex” (ie, many components interacting in a dynamic and unpredictable way). Crucially, NASSS surfaces factors that are often unacknowledged in technology implementation programs, helping to move beyond the identification of individual barriers and enablers in recognition of the dynamic interactions between the domains, for example, the relationship between the individual adopter and the organizational or wider system context.

### Search Strategy

A search strategy was developed in collaboration with an academic librarian. As a preliminary examination of the literature indicated that only a few reviews focused solely on acute care hospitals or older adults and because the specific health care setting was not always immediately clear, we decided not to include filters for population or health care setting in order not to exclude potentially relevant publications.

The full search strategy is shown in Textbox 1. Search terms related to patient portals and systematic reviews were used. The search strategy used a combination of medical subject headings and free-text words. Searches were restricted to 2010 to account for the pace of development in portal technology. Searches were conducted on June 16, 2020, and included the following databases: MEDLINE and Embase via the Ovid platform, CINAHL and PsycINFO via the EBSCO platform, and the Cochrane Library. Reference lists of the included reviews were screened for additional literature.

To generate sufficient breadth of coverage for the scoping review, inclusion criteria were defined to capture maximum learning with respect to the implementation of tethered patient portals among older people and in acute care hospitals. Specifically, 3 categories of reviews were eligible for consideration:

1. Systematic reviews of patient portals in acute care hospitals
2. Systematic reviews of reviews (with both primary studies and reviews) of patient portals in multiple settings, including acute care hospitals
3. Systematic reviews of patient portals for older adults in multiple settings, including acute care hospitals

The inclusion criteria were as follows: reviews published since 2010 in English; reviews focused on the implementation of tethered patient portals (as defined in the *Introduction* section); reviews focused on patient portals for older adults (in settings that include acute care hospitals); reviews focused on patient portals for patients, health care professionals, managers, and budget holders in acute care hospitals; reviews of reviews of patient portals in settings that include acute care hospitals; reviews focused on contextual factors (ie, barriers and facilitators) that impact the implementation of patient portals; systematic reviews; scoping reviews; narrative reviews; qualitative meta-syntheses; meta-ethnographies; and reviews of reviews.

The exclusion criteria were as follows: reviews published before 2010; reviews not in English; reviews not focused on the implementation of tethered patient portals; reviews focused on the technical aspects of patient portals; reviews in family practice settings only; nonsystematic reviews; secondary analyses of the existing data sets; discussions of literature for theory building or critique; summaries of literature for information or commentary; editors’ discussions; letters; conference abstracts; and theses; and reviews whose full text was not available.

**Search strategy by database.**

**Search terms for MEDLINE and Embase (via OVID)**

1. Exp Patient Portals/
2. Health Records, Personal
3. Patient ADJ2 Portal*.mp
4. Electronic ADJ2 Portal.mp
5. (personal adj2 (health or medical) adj2 (record* or info*)).mp
6. Patient accessible record*.mp
7. PHR.mp
8. ePHR.mp
9. or/1-8 (MEDLINE) or/3-8 (EMBASE)
10. Meta analysis/
11. Meta-analysis.ti,ab,pt.
12. Meta-ethno*.ti,ab,pt.
13. Review.ti,ab.pt.
14. or/10-13
15. and/9,14
16. Limits – English Language, 2010-current, humans

**Search terms for CINAHL and PsycINFO (via EBSCO)**

1. MH “Patient Portals”
2. MH “Medical Records, Personal”
3. Patient n2 Portal*
4. Electronic n2 Portal
5. Personal n2 (health or medical) n2 (record* or info*)
6. Patient accessible record*
7. PHR
8. ePHR
9. or/1-8 (CINAHL) or/3-8 (PsycInfo)
10. MH Meta analysis
11. Meta-analysis
12. Meta-ethno*
13. Review.ti,ab.pt.
14. or/10-13
15. and/9,14
16. Limits – English Language, 2010-current

**Search terms for Cochrane Library**

1. Exp Patient Portals
2. Health Records, Personal
3. Health Records, Electronic

### Study Selection

**Figure 1:**
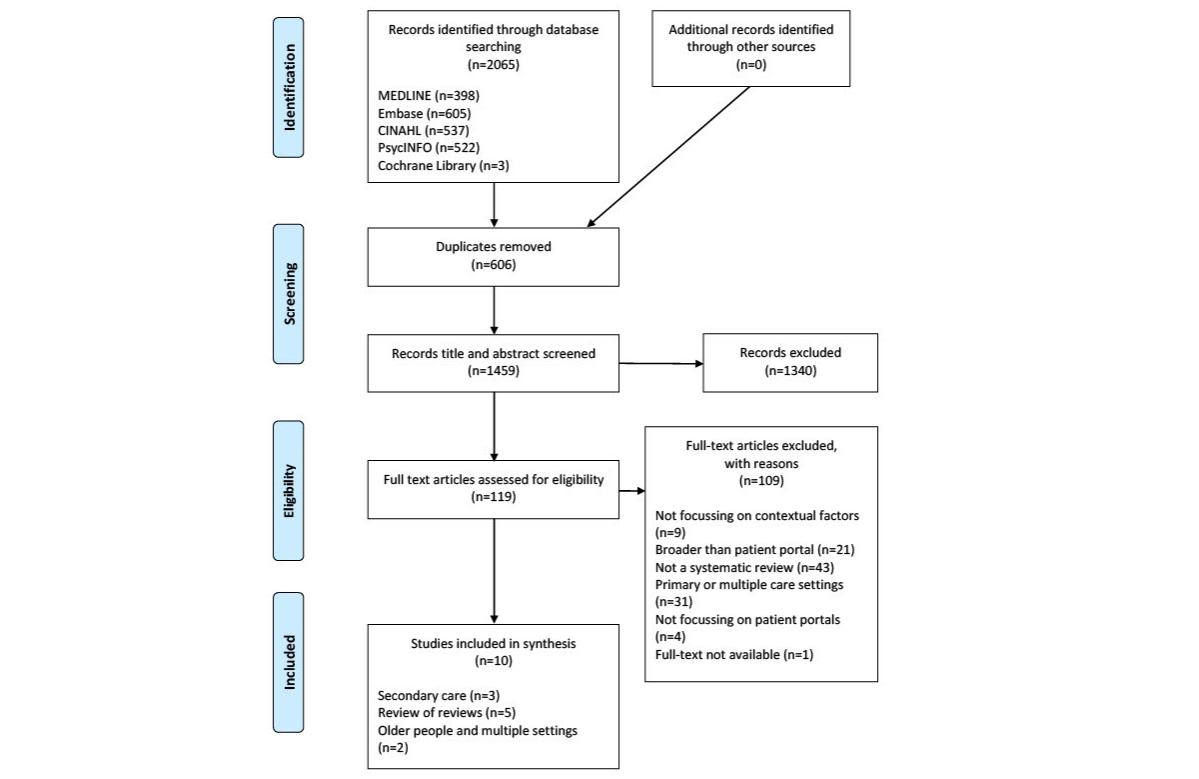
PRISMA (Preferred Reporting Items for Systematic Reviews and Meta-Analyses) diagram.

Search results were imported into EndNote reference management software (Clarivate Analytics), and duplicates were removed automatically and double checked manually. Two reviewers (JH and ZK) independently screened titles and abstracts. Any discrepancies in the articles identified for full-text screening were discussed, and consensus was reached. Full-text articles of potentially eligible reviews were assessed independently by 2 reviewers (JH and ZK) against the prespecified inclusion and exclusion criteria. Discrepancies were resolved through discussion. The reasons for exclusion were recorded and included in the PRISMA diagram (Figure 1).

### Data Charting

In accordance with the Arksey and O’Malley framework for scoping reviews [17], a data charting form was compiled in Microsoft Excel (Microsoft Corp), which contained a row for each included study and columns to record general study information, namely authors, publication date, country of origin, review aim, health care setting, participants, definition of patient portal, theoretical framework, database searches, inclusion and exclusion criteria, data extraction method, quality assessment method, method of analysis or synthesis, and the number of included studies. The included studies were examined to determine the extent of study overlap between the included reviews. The data charting form was also used to extract data on the contextual factors (barriers and facilitators) that impacted the implementation of patient portals in accordance with the 7 domains of the NASSS framework (refer to the *NASSS Framework* section), together with the authors’ recommendations for future research. The form was piloted on 4 studies. A single reviewer (JH) read each study and extracted the study characteristics and data on contextual factors from the results and discussion sections. The discussion sections were included in the charting process, as they often provide additional material to enhance the interpretation of review findings. The data extraction for each of the 4 articles was cross-checked by another team member (TJB, ZK, JL, and FS) to verify whether data charting was performed in accordance with the 7 NASSS framework domains. Data charting was then completed by a single reviewer (JH) and independently verified by another member of the team (ZK).

### Summarizing the Results

A narrative approach was used to summarize the results of the scoping review. In line with the usual practice for scoping reviews [17], no attempt was made to assess the quality of the included reviews or the weight of the evidence with respect to the implementation of patient portals.

## Results

### Search Results

A total of 2065 references were identified (Figure 1). After the removal of duplicates (n=606, 29.35%), another 1340 (64.9%) records were excluded based on the title and abstract, leaving 119 (5.76%) full-text articles to be assessed for eligibility. Of the 119 full papers assessed, 10 (8.4%) met the criteria for inclusion in this scoping review. Scrutiny of the reference lists of the included reviews did not generate additional literature for inclusion. Multimedia Appendix 1 provides details on the characteristics of the included reviews [15,20-28].

### Description of the Included Reviews

The reviews were published between 2015 and 2020. Of the 10 included reviews, 4 (40%) originated from the United States [15,20-22], 2 (20%) each from the United Kingdom [23,24] and the Netherlands [25,26], and 1 (10%) each from Australia [27] and Iran [28]. Overall, 3 (30%) systematic reviews addressed patient portals in acute care hospitals [15,20,23], 5 (50%) systematic reviews of reviews examined patient portals in both acute care hospitals and other care settings [24-28], and 2 (20%) systematic reviews addressed the implementation of patient portals among older adults (in multiple settings, including acute care hospitals) [21,22].

Of the included reviews, 7 (70%) did not specify a particular framework for the analysis of the results [20-24,27,28], 1 (10%) used the System Engineering Initiative for Patient Safety model to categorize interventions [15], 1 (10%) used the Clinical Adoption Framework [25], and 1 (10%) applied the Problem Solving Cycle [26]. Reviews in which no framework was specified considered portal design, use, and usability [20]; input, process, and output factors [27]; content and capabilities [28]; impact on outcome measures [24]; barriers to and facilitators of adoption and user experience [21]; characteristics of older users, evaluation of outcome measures and results, and barriers to and facilitators of use [22]; and impact on trust and communication and consideration of ethical issues [23].

### Study Overlap

To establish the breadth of coverage of this scoping review, an indication of the extent of overlap of studies in the included reviews was determined. The included reviews contained 206 studies (156 primary studies, 75.8%, and 50 reviews, 24.3%), excluding the 109 references of Otte-Trojel et al [26], which, unfortunately, we were not able to obtain for scrutiny. Table 1 provides a summary of the extent of study overlap. Only 19 studies appeared in >1 included review, suggesting limited duplication in the scoping review.

**Table 1:** Overlap of studies^a^.

	Acute care hospitals, n			Multiple settings (review of reviews), n						Older adults (aged >60 years), n	
	D’Costa et al [23], 2020	Grossman et al [15], 2019	Kelly et al [20], 2018	Dendere et al [27], 2019	Aslani et al [28], 2020	van Mens et al [25], 2019	Otte-Trojel et al [26], 2016^b^	Jilka et al [24], 2015	Sakaguchi-Tang et al [21], 2017		Kneale and Demiris [22], 2017
D’Costa et al [23], 2020	N/A^b^	0	3	3	0	0	N/A	0	0		0
Grossman et al [15], 2019	0	N/A	0	1	0	0	N/A	0	0		0
Kelly et al [20], 2018	3	0	N/A	6	0	0	N/A	0	0		0
Dendere et al [27], 2019	3	1	5	N/A	1	6	N/A	2	0		2
Aslani et al [28], 2020	0	0	0	1	N/A	0	N/A	0	0		1
van Mens et al [25], 2019	0	0	0	6	0	N/A	N/A	0	0		0
Otte-Trojel et al [26], 2016^c^	N/A	N/A	N/A	N/A	N/A	N/A	N/A	N/A	N/A		N/A
Jilka et al [24], 2015	0	0	0	2	0	0	N/A	N/A	0		0
Sakaguchi-Tang et al [21], 2017	0	0	0	0	0	0	N/A	0	N/A		1
Kneale and Demiris [22], 2017	0	0	0	2	1	0	N/A	0	1		N/A

^a^Overlap tracked in 156 primary studies and 50 reviews.

^b^N/A: not applicable.

^c^Not able to obtain reference list.

### Narrative of Results by NASSS Framework Domains

#### Overview

The results and discussion sections of the included studies were successfully mapped to the NASSS framework domains and subdomains. The findings for each domain are presented and a summary is provided in Table 2. The headings for the domains and subdomains are taken directly from the NASSS framework. Although each domain is commented on in turn, there are interrelationships between the findings that are highlighted in the text and addressed at the end of this section.

**Table 2:** Non-adoption, Abandonment, Scale-up, Spread, and Sustainability (NASSS) framework: contextual factors that influence the implementation of patient portals.

NASSS domain and subdomain and contextual factors (namely, facilitators and barriers)					Acute care hospitals		Mixed health care setting		Older adults (aged >60 years)
**1. Condition**									
	**What is the nature of the condition or illness?**								
		**Facilitators**							
			Greater disease severity	—^a^		[24]		—	
			Chronic disease (and associated well-established case management programs)	—		[21,23]		—	
		**Barriers**							
			Severity and circumstances of illness (eg, reduced involvement in decision-making and fewer questions)	[20]		[24]		—	
	**Relevant sociocultural factors and comorbidities**								
		**Facilitators**							
			Higher socioeconomic status, female sex, younger age, White ethnicity, and younger senior citizens	—		[23,24]		[18]	
			Disease-specific portal	—		[24]		—	
		**Barriers**							
			Lower socioeconomic status, male gender, older age, and non-White ethnicity (impacts enrollment and engagement)	[12,20]		[24]		—	
			Diversity of older adults (not well understood)	—		—		[19]	
			Low health literacy and numeracy (and understanding of health literacy)	—		[23,24]		[19]	
			Lack of digital access	—		[23]		[18]	
			Insurance status	[12]		—		—	
			Comorbidities such as vision and hearing loss, decreased dexterity and mobility, and declining cognitive function	—		—		[18,19]	
**2. Technology**									
	**Key features**								
		**Facilitators**							
			Information and identity authentication and protection	—		[23,25]		—	
			Usability (eg, set-up, interface design, simple displays, text visibility, buttons, patient-friendly content, ease of navigation, personalized interface, and reminders to view)	[12,17,20]		[21,24,25]		[18]	
			Functionality (eg, communication with providers; access to reliable, timely and comprehensive personal medical information; content in minority languages; and inbuilt system alerts)	[17]		[23,24]		[18]	
			Accessibility (eg, adding mobile access and providing on-site kiosks)	[12]		[21,23]		—	
			Participatory and iterative design approaches	—		[23,24]		—	
			Iterative user evaluation (eg, patients and providers)	[12]		—		—	
			Definition of minimum data set to plan care and continuously evaluate treatment	—		[25]		—	
		**Barriers**							
			Patients’ security and privacy concerns (eg, control over access)	[17,20]		[21-24]		[18]	
			Providers’ concerns about sharing patient information	—		[21,25]		—	
			Usability (eg, interface design, technical glitches, log-on, navigation, accessibility of information for patients, and printing and using information)	[17]		[21,23,24]		[18,19]	
			Establishing a trade-off among security measures, user friendliness, and flexibility	—		[23]		—	
			Functionality (eg, differing information needs of patients and providers; differing patient preferences over data content and input; diversity of health data types and formats and portal ability to handle the diversity of health data types and formats; data transparency—what data are released and to whom and how they are released; language used; and level of features [eg, reminders, dictionary, lifestyle advice, print capability, and user voice command])	[17]		[23-25]		[18]	
			Accessibility (eg, computer and internet access and secure and stable infrastructures)	—		[21-23]		[18]	
	**Type of knowledge in play**								
		**Facilitators**							
			Data set is comprehensive, reliable, complete, understandable, and valid	—		[25]]		—	
			Audit trail for revisions to data	—		[23]		—	
		**Barriers**							
			Concerns about patients’ ability to interpret test results and deal with sensitive information without professional support or interpretation	[17,20]		[21,23,24]		[18]	
			Real-time (release of) information without real-time support	[20]		—		—	
			Providers’ concerns about the reliability of patient-generated data (as basis for clinical decisions)	[20]		[23]		—	
	**Knowledge to use**								
		**Facilitators**							
			Patient training and technical support (eg, videos, handbooks, hotline, and workshops)	[12]		[23]		[18]	
			Training for providers	—		[23]		—	
		**Barriers**							
			Quality of patient training	[12]		—		—	
			Patients’ level of technology literacy (eg, perceived and actual skill and computer anxiety)	[12]		—		[18]	
	**Technology supply model**								
		**Facilitators**							
			Portals that integrate into preexisting systems	[17]		—		—	
			Interoperability (eg, information exchange and sharing)	—		[25]		—	
		**Barriers**							
			Interoperability (eg, achieving appropriate data exchange among systems)	—		[21,23]		—	
**3. Value proposition**									
	**Supply-side value**								
		**Facilitators**							
			Facilitates the processing of payments by insurance companies	—		[25]		—	
			Trial period before purchase (ie, to test usability and estimate financial and organizational impact)	—		[23]		—	
			Positive return on investment and impact on charges and costs	—		[21]		—	
		**Barriers**							
			Trade-off among the type of architecture, responsiveness to local needs, and implementation time and cost (ie, decentralized and more expensive but more responsive and shorter implementation time)	—		[25]		—	
			Establishing sound business case (eg, no standardized evaluation frameworks, no reimbursement structures for electronic services, lack of evidence of cost savings, and lack of financial sustainability)	—		[23,24]		—	
	**Demand-side value**								
		**Facilitators**							
			Satisfies patients’ need for information; facilitates knowledge retention, understanding, and engagement in care by patient; sense of empowerment and control; feeling of being better prepared; and perceived usefulness (eg, aids self-management, utility features, and information in one place)	[17,20]		[21,22,24,25]		[18,19]	
			Provide communication route with professionals between clinic rounds (eg, patient driven communication)	[17]		[21,24]		[18]	
			Assists (verbal) interactions or appointments with professionals and patient-provider communication	[17,20]		[21,22,24,25]		[18]	
			Access to information facilitates the development of trust in diagnosis, investigations, treatment, and professionals (eg, relationships)	[20]		[21]		[19]	
			Helps inaccuracies in EHR^b^ to be identified (eg, detection of errors and patient safety)	[17,20]		[24]		—	
			Contributes to enhanced discussions with patients and aids communication	[17]		[21,24,25]		—	
			Prevents misunderstandings and builds trust (ie, careful and clear recording of information)	[20]		[21]		—	
			Usefulness and time efficiency (ie, clear recording prevents the need to repeat information and aids interprofessional communication)	—		[21,25]		—	
			Helps improve care (eg, planning and continuous evaluation of treatment, adherence, patient satisfaction and engagement, reduced patient anxiety, timely decision-making)	—		[24,25]		—	
		**Barriers**							
			Patients perceive extra responsibility for finding errors or poor outcomes	[20]		—		—	
			Patients’ concern about threat to face-to-face communication with professionals	[20]		—		[18]	
			Patients’ do not see value or usefulness (eg, lack awareness of features)	[12]		[21,23,24]		[19]	
			Patient views about “user fee for use”	—		—		[19]	
			Professionals’ concern that messaging may adversely impact verbal communication	[17]		—		—	
			Professionals do not perceive usefulness	—		[21]		—	
**4. Adopter system**									
	**Changes in staff roles, practices, and identities**								
		**Facilitators**							
			Accepting of collaborative versus expert-led care	[20]		—		—	
			Professionals’ positive level of engagement, knowledge, and confidence in portal systems	—		[24]		—	
		**Barriers**							
			Less accepting of collaborative versus expert-led care and do not wish to cede autonomy to patients	[20]		[23]		—	
			Professionals need to support patients to interpret and emotionally deal with the information in portals	[20]		—		—	
			How is responsibility for the release of test results managed? (eg, who takes responsibility and editing before release)	[20]		—		—	
			Professionals’ level of engagement, knowledge, skills, and confidence in portals	—		[2,21,23]		—	
	**What is expected of patients?**								
		**Facilitators**							
			Professionals support and encourage patients’ use of portals (eg, endorsement, reminders, and materials)	—		[22-24]		—	
			Patients’ willingness to enter basic information into portals or manage records	—		—		[18,19]	
		**Barriers**							
			Patient preferences regarding the entry of data into portals, increased knowledge, and managing records	[20]		[24]		[18,19]	
			Professionals or providers do not encourage patients’ use of portals	—		[23]		—	
	**What is assumed about the network of lay care givers?**								
		**Facilitators**							
			None identified	—		—		—	
		**Barriers**							
			Patients lack help or support to access portals	—		—		[18]	
**5. Organization**									
	**Organization’s capacity to innovate**								
		**Facilitators**							
			Leadership involvement in portal design and development of policies for user training and the integration of patient portals into workflows	—		[24]		—	
			Communication around technical, interpersonal, and workflow aspects of portals	—		[23]		—	
			Organizational interpretation of government legislation related to portals	—		[24]		—	
		**Barriers**							
			Constrained financial context (eg, small or rural hospitals)	[20]		[24]		—	
			Organizational interpretation of government legislation	—		[24]		—	
			Lack of leadership support (fear and hesitancy in implementation)	—		[25]		—	
	**Is the organization ready for technology-supported change?**								
		**Facilitators**							
			Policies in place to support portals (eg, universal access policy, security protocols, adherence audits, data availability, and timing)	[12]		[23]		—	
		**Barriers**							
			Lack of support for new forms of communication between patients and professionals	—		[24]		—	
			Lack of policies on access rights and authorization process (including proxy access and access for minors)	—		[23]		—	
	**Ease of funding and adoption decision**								
		**Facilitators**							
			Internal and external exchange of information to improve the quality, safety, and effectiveness of care	—		[25]		—	
		**Barriers**							
			Providers’ concerns about diverting resources to the less disadvantaged (ie, those who can read and ask questions)	[20]		—		—	
			Integrating patient portal use across care transitions (ie, with other organizations) to improve care	[17]		[25]		—	
			Deciding on the balance between IT structure and implementation time and cost			[25]			
	**Changes needed in team interactions and routines**								
		**Facilitators**							
			Integrating data release with workflow (ie, to facilitate professionals’ follow-up with patients)	—		[23]		—	
			Workload and work routines not adversely impacted or positively impacted (eg, time efficiencies)	—		[21]		—	
		**Barriers**							
			How to organize the release of results to patients without professionals’ help with interpretation and support (eg, real-time release or delayed released)	[20]		—		—	
			Professionals’ concerns about the impact of increased level of patient questions, patient overuse of messaging, increase in documentation time, and portals on workflow	[17]		[21,23,24]		—	
	**Work involved in implementation and who will do it**								
		**Facilitators**							
			Involvement of professionals in workflow engineering and the evaluation of the impact of portal use on workload and processes	[17]		[23]		—	
		**Barriers**							
			None identified	—		—		—	
**6. Wider context**									
	**What is the political, economic, regulatory, professional, and sociocultural context of program rollout?**								
		**Facilitators**							
		Aspects of culture (doctors from English-speaking countries), including the coverage of portals, PHRs^c^, and EHRs in medical and nursing school curricula		[20]		[23]		—	
		Health professionals’ liability concerns		[20]		—		—	
		Health systems with a transactional component		[20]		—		—	
		Resource for policy makers, health care specialists, and stakeholders to improve care and the quality of treatment		—		[25]		—	
		National and international information exchange (interoperability) and other standards (eg, Health Insurance Portability and Accountability Act, International Health Level 7, regional health information exchanges, and key public infrastructures)		—		[23,25]		—	
		Appropriate reimbursement mechanisms		—		[23]		—	
	**Barriers**								
		Reimbursement structures for electronic services		—		[23]		—	
		Providers’ liability concerns (eg, breached privacy or patients’ harmful behaviors)		—		[23]		—	
		Nonstandardized rules for developing and managing health information infrastructures		—		[23]		—	
		Relationship between macrolevel and mesolevel (eg, organization) factors was not well explored		—		[22]		—	
		Regulations (eg, Health Insurance Portability) do not cover portal developers and hosting organizations		—		[23]		—	
**7. Embedding and adaptation**									
	**Scope for adapting and coevolving technology and service**								
		**Facilitators**							
			None identified	—		—		—	
		**Barriers**							
			Concern that medical record maintains integrity as a working document that facilitates the transfer of knowledge between health professionals	[20]		—		—	
			How portals can be extended beyond a single organization (ie, particularly in fragmented care delivery contexts)?	—		[23]		—	
	**Organization resilience to critical events**								
		**Facilitators**							
			None identified	—		—		—	
		**Barriers**							
			None identified	—		—		—	

^a^Not available.

^b^EHR: electronic health record.

^c^PHR: personal health record.

#### Domain 1: The Condition

##### What Is the Nature of the Condition or Illness?

The included reviews documented patient portals that are open to individuals with a variety of health conditions, including acute and chronic diseases and high-risk conditions. Reviews focusing on patient portals in acute care hospitals included adult patients with acute medical conditions [23], inpatients and outpatients classified as vulnerable (including those with cardiovascular diseases, those with HIV, those with ophthalmic conditions and those with chronic or unspecified conditions) [15], patients who have had cardiac surgery, patients who were in an intensive care unit, patients with cancer, parents of patients who were in a neonatal intensive care unit, and caregivers or patients who underwent bone marrow transplant. Reviews focusing on multiple care settings included patients with multiple sclerosis [28], patients in any medical domain [25], adult patients with chronic diseases, patients in family practice settings [24], and patients with unspecified conditions [26,27]. The reviews focused on older patients aged ≥60 years did not specify an illness or medical domain.

There was limited consideration of how clinical characteristics played a role in patient portal use. An examination of inpatient portals [27] highlighted patients’ desire to be able to view their daily schedule, view information on medications and test results, and learn about care and preparations for discharge. This review acknowledged how the nature of an individual’s condition (eg, severe illness or intense pain) could affect their ability or desire to interact with the functionality of a patient portal, as well as their capacity to be involved in decision-making about their care and to formulate and ask questions to health professionals [23,27].

A total of 3 (30%) reviews suggested that patients with greater disease severity [27] or with chronic disease [24,26] may engage more with a portal. Patients with chronic diseases (such as diabetes, hypertension, or depression) have the benefit of well-established case management programs [24]. Although this may facilitate the adoption of a portal, the authors noted that concomitant case management programs will also be a factor impacting disease outcomes (separate from any impact on patient outcomes from the portal), making the findings of disease-specific studies of patient portal implementation difficult to extrapolate across non–disease-specific populations [24].

##### What Are the Relevant Sociocultural Factors and Comorbidities?

The NASSS framework considers how complexity occurs when a condition or an illness is associated with sociocultural factors (eg, poverty or social exclusion) and comorbidities (eg, loss of function and multimorbidity of older age). The impact of these factors on patient portal adoption received more extensive coverage in the included reviews, along with concerns about the potential for the exacerbation of health inequalities owing to disparities in engagement, as outlined subsequently.

The included reviews suggested that portal adoption is associated with having a higher socioeconomic status, being female, being of White ethnicity, being younger, and being a younger senior citizen [21,26,27].

Low health literacy and numeracy in patient groups [21,22,27] as well as a lack of digital access [21,26] were identified as barriers to portal use. Vulnerable groups [15], those with lower socioeconomic status [15,23,27], and those with less favorable health insurance status [15] are less likely to be enrolled in or engage with patient portals. In addition, increasing age, male sex, and non-White ethnicity were identified as factors associated with low adoption [15,23,27].

Certain comorbidities, such as vision and hearing loss, decreased dexterity and mobility, and declining cognitive function, were identified as barriers to portal use [21,22]. These factors may be associated with the aging process; however, 1 (10%) review highlighted that the diversity of older adults and their needs relative to patient portals are not well understood [21].

#### Domain 2: The Technology

##### Material and Technical Features

The included reviews did not outline the technology in detail but identified several material and technical features that promote patient portal adoption and user satisfaction, including a well-designed interface [20,28], ease of setup and access [21], straightforward navigation [20] and information transfer [21], simple formats [20,23] and buttons [23], text visibility [28], user-friendly content [20], error messages [28], real-time [20] or disease-specific information [27], email reminders to view content [15], and a personalized interface [20].

Conversely, poor usability features, such as poor interface design [24,26,27], technical glitches [27], log-on [21] or navigation difficulties [21,22], and difficulties with printing and using information [22] were reported to have a negative impact on users’ experience, and satisfaction, with patient portals [20-22,24,26,27]. Moreover, 1 (10%) review suggested that if patients perceive the access to their personal health records as useful, they are more willing to overcome the technical barriers of engaging with the patient portal [22]. This is linked to domain 3 (the value proposition) in terms of the desirability or value of the portal technology for patients.

The reviews suggested that participatory and iterative design [26,27] and iterative user evaluation [15], including both patient and health professional users, at the planning and development stage of a patient portal are ways to overcome usability issues. Such inclusive and consultative design approaches also allow the functionality of patient portals to be addressed [20,26-28]. Reviews reported that patient engagement with portals can be facilitated by offering desired features, including communication with health professionals [20]; access to reliable, timely, and comprehensive personal medical information [27]; and content in minority languages [26]. The reviews also pointed to the benefits for health professionals from their involvement at the design stage, including the specification of a minimum data set for care planning and for the continuous evaluation of treatment [28], and from inbuilt system alerts (eg, if a patient does not open an email or to signal a medical emergency) [26].

Several reviews highlighted potential difficulties in defining the functionality of a patient portal system, including the differing information needs of patients and health professionals [20], differing patient preferences regarding data content and data input [27], the diversity of health data formats [28], the language to be used (ie, designing content for lay and professional audiences) [26], the level of features (eg, reminders, dictionary of medical terms, lifestyle advice, and print and user voice command capability) [21], and data transparency (ie, deciding what data to release to patients and when and how to make them available) [26].

The information privacy and security aspects of patient portals were reported to be an area of concern. This could be patients’ concerns regarding their personal health information [20,21,23-27] or health professionals’ concerns about sharing patient information [24,28]. Measures such as robust identity authentication and information protection [26,28] were suggested as mechanisms to address such concerns, with the observation that there can be a trade-off between security measures and user friendliness [26].

Accessibility of the technology for patients [24], including computer [21] and internet [21,25] access, was highlighted as another barrier to the implementation of patient portals. This may be because of cost issues [21]. Suggested mechanisms to promote accessibility were making mobile as well as computer access available [15] and providing on-site kiosks [26]. Establishing secure and stable technical infrastructures on which portals can operate was reported to be a challenge for providers [26].

##### Type of Data Generated or Knowledge in Play

In terms of the data held in the patient portal, 1 (10%) review suggested that the data set needed to be comprehensive, reliable, complete, understandable, and valid [28], with another (10%) recommending the inclusion of an audit trail so that the revisions made to the data are visible [26].

Several reviews raised the issue of health professionals’ concerns about patients’ access to health information via portals, particularly sensitive information, with questions about how patients can deal with the information without professionals’ help with interpretation and support [20,21,23,24,26,27]. This concern particularly revolved around the issue of the real-time release of data or test results without real-time support [23] and is linked to domain 5 (the organization), whereby health professionals need to adapt to patient portal technology and incorporate it into their practice. Patient portals providing opportunities for patients to enter data about their condition raised additional questions about knowledge in play; 2 (20%) reviews reported that health professionals can have reservations about the reliability of patient-generated data in a portal and whether these data should form the basis of clinical decisions [23,26].

##### Knowledge Needed to Use

The reviews suggested that training and support can help portal use [15,26] and the use of specific features [15], helping to overcome the barrier of patients’ technology literacy [15,21,26], including perceived and actual computer and internet skills [21]. However, 1 (10%) review reported that it is possible for training to have unintended consequences (ie, decreased intention to use) [15]. The training of health professionals must also be addressed [26]. It was posited that various tools and aids can facilitate the understanding of portal concepts and navigation, health information, and health management tasks (eg, videos, user handbooks, hotlines, and workshops) [21].

##### Technology Supply Model

Although the included reviews did not address how the patient portal technology was procured, the lack of interoperability for achieving appropriate data exchange between systems was identified as a barrier to adoption [24,26]. Portals that can be integrated into preexisting systems or offer data sharing and exchange are identified as facilitators [20].

#### Domain 3: The Value Proposition

##### What Is the Developer’s Business Case for the Technology (Supply-Side Value)?

The reviews did not address the issue of the business case for patient portals from the developer’s perspective but did examine it from the health care system’s point of view, primarily with respect to the difficulties in establishing such a case [23,24].

Uncertainty around cost savings and financial sustainability, as well as reimbursement models for electronic services [26], contributes to complexity in this situation. The absence of standardized evaluation frameworks means that evidence of benefits (such as administrative efficiency or better-managed patients) is lacking. In addition, 1 (10%) review highlighted the challenge of deciding on a balance between technology architecture (ie, centralized or decentralized), its responsiveness to local needs, ease of implementation, and cost when compiling a business case; decentralized architectures are reportedly more compatible with local needs and can be implemented more quickly but have higher costs [28].

A recommendation for ensuring a sustainable business case was to have a trial period before committing to the purchase of a portal [26]. This allows the testing of usability and provides an opportunity to estimate the likely financial and organizational effects [26], such as the facilitation of the processing of payments [28]. It was suggested that determining a positive return on investment and the potential for lower hospital costs will support implementation [24].

##### What Is the Desirability, Efficacy, Safety, and Cost-effectiveness (Demand-Side Value)?

The reviews suggested that patient portals do satisfy patients’ need for information (eg, about hospital schedule, medication, test results, and discharge planning) [20-22,27,28], helping with knowledge retention [23] and interactions with professionals [20,21,23-25,27,28] and providing a communication channel between clinic rounds [20,21,24,27]. These features support patients’ understanding of their condition [22,24,25] and engagement in care or self-management [21,22,24,25,27], leading to a greater sense of empowerment and control [25,27] and feeling of being better prepared (ie, for appointments, emergencies, and discharge) [20,21]. In addition, the reviews indicated that access to information via the portal also facilitated the development of trust in health professionals [22-24], with patients feeling reassured by shared information [23].

Professionals valued patient portals as a mechanism for enhancing patient care [27] through timely decision-making [28], planning and continuous evaluation of treatment [28], and building trust [23,24] and as a mechanism that leads to improved patient engagement, adherence to treatment, and satisfaction with care [27,28]. Portals are also regarded as an aid to communication with patients [20,24,27,28] and as a tool to enhance interprofessional communication [24,28]. The clear recording of data in a portal was found to help efficiency by reducing the need to repeat information [24,28] and to contribute to patient safety by allowing inaccuracies and errors to be identified [20,23,27].

As indicated under domain 2 (the technology), where patients perceive portals as useful, they are prepared to overcome the technical barriers to portal use [22]. However, some reviews identified a lack of perceived usefulness from the patient perspective as a barrier to engagement with a portal [15,22,24,26,27], together with patient views about “fee for use” [22]. Some patients regarded portals as a threat to valued face-to-face communication with health professionals [21,23] or felt an additional (and unwelcome) burden of responsibility with respect to their care (eg, for finding errors or for poor outcomes) [23]. Some health professionals also did not see the usefulness or value of patient portals [24] or felt that they would adversely impact face-to-face communication with patients [20].

#### Domain 4: The Adopter System

##### What Changes in Staff Roles, Practices, and Identities Are Implied?

The reviews highlighted that the adoption of patient portals can raise questions regarding health professionals’ scope of practice and professional identity. There are implications in terms of health professionals’ confidence and ability to interact with the technology [24,26,27]; their need to incorporate the technology into their work practices [23]; and the potential for patient portals to alter the balance of the professional-patient relationship, shifting to more collaborative, rather than expert-led, working [23,26]. These elements are linked to considerations in domain 2 (the technology) regarding the involvement of health professionals at the portal design stage, as well as the provision of training and ongoing support for portal use, and to domain 5 (the organization) regarding the potential impact of patient portals on the workflow and workload of professionals and models of care and service delivery.

Moreover, 1 (10%) review suggested that when health professionals advocated collaborative working with patients and had confidence in using patient portals, this acted as a facilitator of implementation [23]. Conversely, where professionals were reluctant to cede professional autonomy and work more collaboratively with patients [23,26] or had concerns about their capacity and skills to engage with portal technology [24,26,27], this acted as a barrier to the implementation of patient portals. Examples of implications for practice included being able to support patients to interpret and emotionally deal with the information contained in the portal and deciding who takes responsibility for the release of information into the portal and whether the information needs to be edited before release [23].

##### What Is Expected of the Patient (or Immediate Caregiver) and Is This Achievable by, and Acceptable to, Them?

Professionals’ support and encouragement of patients’ use of portals were identified as mechanisms to facilitate the adoption of portals among patients [25-27]. Both (20%) the reviews that focused on older adults suggested that patients may be willing to enter basic data into the portal [21,22]. Patient engagement with portals is impacted by different preferences: some patients may not wish to have the responsibility of increased knowledge afforded by the portal [23] and do not wish to enter data [21,22,27] or be responsible for managing records [21].

##### What Is Assumed About the Extended Network of Lay Caregivers?

The included reviews did not directly address expectations of the involvement of the wider care network or lay caregivers in the adoption of patient portals, although it is acknowledged that older patients may lack help or support to access a portal [21]. This subject is linked to the information security and privacy concerns raised in domain 2 (the technology) and to the questions about policies on access (including proxy access) and security in domain 5 (the organization).

#### Domain 5: The Organization

##### What Is the Organization’s Capacity to Innovate?

The included reviews highlighted the importance of organizational leadership support in promoting portal adoption [27,28] through actions such as working with developers on portal design [27] and developing policies for user training (both patients and health professionals) and integrating portals into clinical workflows, as well as organizing communication around the technical, interpersonal, and workflow aspects of patient portals [28]. A lack of executive leadership support can lead to hesitancy with portal implementation [28].

One (10%) review pointed to the potential for variability in portal implementation (eg, content made available to patients) in situations where providers have the discretion to interpret government legislation [27]. This is connected with the issue of internal and external information exchange; domain 6 (the wider context); and the extent to which there are standardized, nationally mandated regulations for developing and maintaining health information technologies. This may also influence the value proposition for patients (domain 3).

A constrained financial context will impact the implementation of portal technology [23,27]. One of the included reviews highlighted resource constraints at small or rural hospitals [27] as a situation likely to make the adoption of patient portals more difficult.

##### How Ready Is the Organization for Technology-Supported Change?

The included reviews highlighted the range of preparatory work that organizations need to do to support portal implementation. Organizational policies such as universal access [15]; security protocols, including those related to proxy access and access for minors [26]; adherence audits [26]; and data availability and timing will facilitate portal development and implementation. One (10%) review pointed to the necessity for sufficient organizational support for new forms of communication between patients and health professionals afforded by patient portals [27].

##### How Easy Will the Adoption and Funding Decision Be?

The challenges of making decisions on adoption and funding were highlighted by the included reviews. Organizations need to decide on the balance between costs, implementation time, and the flexibility of the portal architecture [28], including the ability to integrate portal use across care transitions (ie, interoperability with other organizations) [20,28]. There may be concerns that portals divert scarce resources to those who are *less* disadvantaged (ie, those who can read and have the confidence to ask questions) [23]. However, enhanced communication through internal and external exchange of information may offer positive advantages for the quality, safety, and effectiveness of patient care [28].

##### What Changes Will Be Needed in Team Interactions and Routines?

Concerns among health professionals regarding the potential impact of patient portals on workload and workflow were identified in the reviews [20,24,26,27], including the possibility of an increased level of patient questions [20], the potential for patient overuse of portal messaging functions [20], the question of how to respond to patient inquiries in a timely manner [26], and an increase in documentation time [24]. A related concern was how to manage and organize the release of results to patients without the presence of a health professional to offer help with interpretation and support [23].

Some solutions addressing workflow concerns were presented, including integrating data release to patients with workflow patterns to facilitate health professional follow-up with patients when the results are made available [26] and providing evidence for a positive impact on workflow and workload (eg, time efficiencies) [24].

These issues are linked to the involvement of health professionals at the technology design stage (domain 2), where concerns about the potential impact on workflows can be raised, and to the points raised earlier about the development of policies around integration by organizational leadership and proactive communication around the integration of portals into workflows.

#### Domain 6: The Wider Context

##### What Is the Political, Economic, Regulatory, Professional, and Sociocultural Context for Program Rollout?

The included reviews suggest the development of national and international information exchange (ie, interoperability) and other standards (eg, security) as a facilitator of portal implementation [26,28].

One (10%) review identified health professionals’ liability concerns as a factor that will promote patient access to records (eg, in countries such as Norway and the United States) [23]. For countries with health systems that have a transactional component (eg, the United States), it is posited that portals can act as a mechanism for helping patients understand their health care costs and that this will encourage the provision of the technology [23]. Other cultural components identified as important for adoption included the coverage of portals in medical and nursing school curricula [26] and the perceived benefit of portal data sets as a resource for policy makers, health care specialists, and stakeholders to evaluate and improve care [28].

Barriers to portal implementation identified in the reviews included nonstandardized rules for developing and managing health information infrastructures (ie, for interoperability) and regulations for data protection and management (eg, Health Insurance Portability and Accountability Act in the United States) that do not cover portal developers or hosting organizations, creating uncertainty about appropriate data governance [26]. In addition, it was pointed out that providers may have liability concerns about privacy breaches or patients’ harmful behaviors [26].

One (10%) review pointed to inadequate or contradictory reimbursement structures for electronic services as a wider contextual barrier to the implementation of patient portals [26], inhibiting the development of a sound business model (link to domain 3). This review cited the Meaningful Use program in the United States as an example of a national initiative for patient portal adoption that was hampered by modest incentives and high thresholds, which impeded the development of an adequate business case [26].

#### Domain 7: Embedding and Sustaining

##### How Much Scope Is There for Adapting and Coevolving the Technology and Services Over Time?

In the included reviews, there was little consideration of the long-term adaptability and sustainability of the patient portals. The focus of attention was on development and short-term implementation issues.

Two long-term considerations were mentioned in the literature. The first was a general concern that the medical record maintains its integrity as a working document that facilitates the transfer of knowledge among many health professionals [23]. The second was related to the fact that most portals are implemented within a single organization or organized care delivery system, which limits their relevance to other organizational contexts [26]. Portal implementation will be more challenging across organizational contexts or in fragmented care delivery contexts, which are situations that are likely to feature in older people’s care.

##### How Resilient Is the Organization to Handling Critical Events and Adapting to Unforeseen Eventualities?

There was no coverage of organizational resilience to critical or unforeseen events and ability to adapt to them.

### Coverage and Interactions Between NASSS Domains

Table 2 shows that the contextual factors influencing implementation identified in the included reviews tended to cluster in specific domains: (1) the condition, (2) the technology, and (3) the value proposition. Certain aspects within these domains received more coverage than others, such as sociocultural factors and comorbidities, the usability and functionality aspects of the technology, and the demand-side value. The included reviews that used a theoretical framework [15,26,28] pointed to a focus on a narrow range of components of patient portal adoption, usually people and use factors.

There are links among the different domains. For example, the severity of an individual’s illness can affect their ability to interact with portal technology, raising questions about expectations of the involvement of lay caregivers (domain 4: the adopter system), organizational policies on proxy access (domain 5: the organization), privacy and security features (domain 2: the technology), and information governance (domain 6: the wider context). Similarly, organizational leadership and support (domain 5: the organization) for the development and implementation of portals can ensure inclusive and iterative portal design (domain 1: the technology), addressing not only usability and functionality issues but also the perceived value (domain 3: the value proposition) of the technology, as well as concerns about the impact of portals on health professional roles and identities (domain 4: the adopter system) and workload and workflow (domain 5: the organization).

There are gaps in the literature pertinent to the consideration of the provision of patient portals among older people in acute care hospitals, including the lack of consideration of the diversity of older adults and their needs, the question of interoperability between systems (likely to be important where care involves multiple services), the involvement of lay caregivers and looking beyond short-term implementation to ways in which portal use can be sustained.

## Discussion

### Summary of Key Findings

This scoping review provides an overview of the contextual factors that impact the implementation of patient portals through an exploration of the emerging literature on patient portal use and engagement in acute care hospitals and among older people. Patients with chronic disease or greater disease severity were found to engage more with portals; however, comorbidities associated with the aging process were identified as barriers to portal use (domain 1: the condition). Perceived benefits from the supply side supported the adoption of portals, such as the potential for lower hospital costs, as did benefits from the demand side, such as engagement in care or self-management (domain 3: value proposition). Training for patients and staff should address technology literacy, the use of portal features, capacity-related concerns (integration of portals into workflows), and perceived value among health care professionals (domain 2: the technology). Older patients may lack help or support to access a portal; however, expectations of the involvement of lay caregivers in the adoption of patient portals were not reported (domain 4: the adopter system). Organizational leadership facilitates portal adoption, such as working with developers on portal design, developing policies for user training, and integrating portals into clinical workflows (domain 5: the organization). The development of national and international information exchange (ie, interoperability) and other standards (eg, security) was as a facilitator of portal implementation within the wider context (domain 6: the wider context). The reviews did not report on the long-term adaptability or sustainability of patient portals or organizational resilience. There were concerns that most portals are implemented within a single organization and that implementation across organizational contexts or in fragmented care delivery contexts would be challenging. This is important for the care of older people (domain 7: embedding and sustaining).

### Older People and Inequalities

The diversity of older adults and their patient portal needs are not well understood. Older patients are more likely to experience chronic disease or greater severity of disease, and patients with chronic illness and greater severity of disease were found to engage more with portals owing to the perceived benefits of self-management, empowerment, and enhanced patient care. However, comorbidities related to age, such as vision and hearing loss, decreased dexterity and mobility, and declining cognitive function impede portal use. Variability in portal use and engagement among older people will, in part, be linked to the reasons for variability in internet use. Low income is the largest impediment to internet use among older people, followed by being aged >75 years, living alone, mobility, and memory or concentration problems [29]. This scoping review found that lower socioeconomic status, increasing age, male sex, and non-White ethnicity were factors associated with low adoption. People of lower socioeconomic status, older people, and people with mobility and memory or concentration problems are regular users of acute care services, making it an ideal setting to address these inequalities in patient portal access and engagement. Training programs and other engagement activities must directly target these inequalities to prevent any unintended exacerbation of the gray digital divide caused by the introduction or widespread use of a patient portal.

### Adopter System

An important gap in the literature identified by this review was the lack of consideration of the involvement of the wider care network or lay caregivers in the adoption of patient portals. Many impediments to internet use among older people are linked to the increased likelihood of receiving care, for example, older age, mobility, and memory or concentration problems [29]. Older people are found to value proxy access to patient portals [30], with motivators including help to manage care, in the event of an emergency and lack of technology experience [31]. However, older patients express concerns when portals contain access to stigmatized conditions and financial commitments [32]. A review of 20 US health systems found that half of them had proxy access functionality, although only a few allowed the patient to specify role-based privileges [33]. The provision of separate proxy access should be accompanied by the provision of more control for patients over the information they wish to share [34]. This review found that organizational policies such as universal access; security protocols, including proxy access; adherence audits; and data availability and timing will facilitate portal development and implementation. To further aid the organizational readiness for technology-supported change, wider contextual factors must be considered at the planning stage in the form of policy shifts and patient developer specifications regarding the facility for internal and external information exchange. There is a need to engage with new ways of managing and talking about people’s data, which may require a different skill set, that is, different stakeholders round the table.

### Technology Supply Model

None of the included studies addressed the procurement process for patient portals. In the United Kingdom, there are a handful of providers that offer patient portals to acute care hospitals, such as Epic and Cerner. Patient portals differ in the extent to which they provide an off-the-shelf product or a tailored product with features that can be switched on or off depending on organizational readiness and capacity to facilitate them, such as patient-clinician communication. This review found that decentralized architectures were more compatible with local needs and implemented more quickly but were associated with higher costs [28]. Furthermore, there is no information on the level of support provided by the technology suppliers for the use of their products. This is anticipated to be a major organizational level barrier to implementation, which needs to be promptly addressed to facilitate the scale-up of portal use in acute care hospitals across the United Kingdom. Portals that can integrate into preexisting systems or offer data sharing and exchange were identified as facilitators [20]. Interoperability of health and care systems and other community services, such as the police and social services, is placed high on the NHS agenda [5], but although organizations may desire data sharing among themselves, the loss of control over shared data may serve as a barrier to portal adoption and highlights the complexity of this approach.

### Strengths and Limitations

This scoping review used a comprehensive set of search terms to identify literature from electronic databases and followed robust procedures for citation and full-text screening in duplicate. Study overlap is a recognized limitation of reviews of reviews, where the primary studies may be reported in >1 systematic review and hence findings are overemphasized. This review included 156 primary studies and 50 reviews. We found that 19 (9.2%) of these 206 studies appeared in >1 review, indicating that study overlap was minimal, although we did not track overlap in the reference lists of all the included reviews. The NASSS framework provides a semantic structure by which to explore multilevel contextual factors impacting the implementation of digital health interventions. NASSS has largely been used to predict and evaluate implementation programs, but more recently, the framework has been used to synthesize review findings [35,36].

The number of reviews that focused exclusively on acute care hospitals (3/10, 30%) and older people (2/10, 20%) was low, which led to a broader scope of the extensive literature, primarily conducted in family practice and other patient groups, to capture learning and potential generalizability of the findings across settings and patients. In broadening the scope of the review, there were similarities with the umbrella review conducted by Antonio et al [14], who used a similar search strategy and a knowledge translation tool to present their findings. Our review was designed and our searches were conducted before the publication of the review conducted by Antonio et al [14]. We believe these similarities reinforce our robust approach to reviewing and synthesizing the literature, particularly as our design aimed to scope and map the literature rather than to systematically review it. The key differences between the reviews include our focus on older people and acute care hospitals; our review design aimed to scope rather than systematically review and appraise the literature; and our application of the NASSS framework. The NASSS has been referenced in >70 JMIR published studies since its publication in 2017, enabling easy comparison with the wider literature.

Papers were selected in accordance with our definition of a patient portal, and we were guided by the authors’ description of a patient portal. Multimedia Appendix 1 shows the definition of patient portals in each of the included studies. The use of the NASSS framework is concerned with the complexity of the use of portals; therefore, all data were considered according to the framework to produce a “big picture” aggregation of what is known about the implementation of patient portals. The included reviews were limited to those published in English; however, we did not exclude reviews that included non-English studies.

### Recommendations for Research

There is substantial literature on the contextual factors impacting patient portal use, with approximately 200 studies identified by the included reviews. However, there are few studies that evaluated interventions to address disparities in the use of patient portals [15]. As highlighted in a systematic review on the implementation of complex interventions in family practice [37], implementation studies exploring contextual factors tend to focus on surveys and qualitative studies, which are valuable in providing individual stakeholder perspectives but need triangulation with other methods. Observation and document analysis should accompany interviews to capture a more complete picture of the contextual factors at play, in particular, the wider context. As with any study exploring or evaluating the determinants of implementing a complex intervention, the features and functionality of the portal should be described in detail using established guidance [38] to enable reflection on the transferability of the findings to other settings. Finally, where interviews are used to explore the determinants of implementation among patients and staff, portal use data could be used to prompt further examination of use and sustained use.

### Recommendations for Practice

This review provides implications for portal adoption and implementation that can inform current practice. This review found that cost, interoperability, trialability, and adaptability were all facilitators of portal adoption. Among hospitals in England deciding which portal product to adopt, GDE trusts play an important role in sharing detailed journeys through a digital technology via GDE blueprints, which are intended to promote scale-up, spread, and sustainability. To maximize the impact of GDE blueprints, they must be reported in an honest and transparent manner, with details on the challenges as well as the benefits of portals’ adoption, engagement, and sustained use. Descriptions of portal implementation must clearly delineate implementation strategies, such as detailed information on training for staff and patients (ie, content, frequency, and format) and communication strategies for the technical, interpersonal, and workflow aspects of patient portals. NHS Digital has created a Personal Health Records adoption toolkit, which offers generic support to organizations looking to implement a patient portal [39]. Furthermore, GDE trusts act as buddy sites to support other trusts, known as “Fast Followers,” for example, by sharing software, IT teams, standard processes, and could possibly assist with the selection and implementation of a patient portal; this approach is a powerful knowledge mobilization strategy enabling successful models to be scaled-up across the NHS [4].

### Conclusions

This scoping review found that contextual factors influencing patient portal implementation tended to cluster in specific domains: (1) the condition, (2) the technology, and (3) the value proposition. Certain aspects within these domains received more coverage than others, such as sociocultural factors and comorbidities, the usability and functionality aspects of the technology, and the demand-side value. There are gaps in the literature pertinent to the consideration of the provision of patient portals for older people in acute care hospitals, including the lack of consideration of the diversity of older adults and their needs, the question of interoperability between systems (likely to be important where care involves multiple services), the involvement of lay caregivers, and looking beyond short-term implementation to ways in which portal use can be sustained. There is substantial literature on the contextual factors impacting patient portal use. Future research should focus on evaluating strategies that address disparities in use and promote engagement with patient portals among older people in acute care settings.

## Appendix

Multimedia Appendix 1 Characteristics of included reviews.

## Abbreviations

GDE: Global Digital Exemplar
NASSS: Non-adoption, Abandonment, Scale-up, Spread, and Sustainability
NHS: National Health Service
PRISMA-ScR: Preferred Reporting Items for Systematic Reviews and Meta-Analyses Extension for Scoping Reviews
