# Supplementary material for: Contextual Factors That Impact the Implementation of Patient Portals With a Focus on Older People in Acute Care Hospitals: Scoping Review
Source: JMIR Aging. 2023 Feb 3;6:e31812. doi: 10.2196/31812 (PMC9938437; doi:10.2196/31812)
Supplement: Multimedia Appendix 1 [file aging_v6i1e31812_app1.docx]

**Multimedia appendix 1: Characteristics of included reviews (part 1)**

| **Reference** | **Country of origin** | **Aim** | **Setting** | **Participants** | **Definition of patient portal** | **Theoretical framework** |
| --- | --- | --- | --- | --- | --- | --- |
| D’Costa et al (2020) | UK  9 studies = USA,  1 each = Israel, Norway & Canada | Identify studies of sharing records with medical patients in inpatient setting, impact on trust & communication, ethical issues | Acute medical care setting (up to 5 days inpatient stay) | Adult medical patients | An electronic route to targeted parts of the medical record | None stated |
| Grossman et al (2019) | USA  Geographical location of included studies not specified | What interventions impact portal use or predictors of portal use in vulnerable populations, what interventions impact disparities in portal use? | Outpatient and inpatient secondary care (academic, safety net & veterans’ hospitals) | Patients in vulnerable patient population (defined by PROGRESS-Plus criteria) | Secure website offering 24-hour access to records, as well as appointment scheduling, medication monitoring and other health management features | System Engineering Initiative for Patient Safety (SEIPS) model to categorise interventions |
| Kelly et al (2018) | USA  Geographical location of included studies not specified | Describe emerging literature on design, use and impact of PP for hospitalised patients and/or caregivers over last 10 years | Hospital Inpatient setting | Inpatients, caregivers, parents, volunteers, healthcare professionals (nurses, doctors, pharmacists, researchers), advisory council members | Inpatient portals are ePP tethered to EHRs that are designed to provide hospitalised patients and/or caregivers secure access to personalised, inpatient clinical information with the intent of engaging them in their hospital care | Studies analysed and described according to 3 themes: 1) inpatient portal design, 2) inpatient portal use & usability, and 3) impact of inpatient portal use on inpatient or caregiver or healthcare team outcomes |
| Dendere et al (2019) | Australia  Geographical location of included studies not specified | Review literature on PP tethered to EMR in inpatient settings & role in patient engagement, impact on HC delivery, factors & best practice for successful implementation, areas for further research | Hospital Inpatient setting | Patients, caregivers, and health care teams | PP tethered to EMR in inpatient setting | No specific framework, studies categorised by input, process and output factors |
| Aslani et al (2020) | Iran  Majority articles published in US (19/39) | Determine the attributes (content and capabilities) of web-based PHRs for MS, collection and categorisation of experiences from different countries to assist policy makers and authorities in implementing and using PHRs to improve conditions of MS patients | Setting not specified | Patients with MS, healthcare providers | PHR an electronic or non-electronic record through which individuals can access, manage and share health records. 3 main architectures of PHRs: tethered, integrated and stand-alone. Focus of review is on web-based PHRs for MS | No specific framework, studies categorised by content (minimum dataset) and capabilities of web-based PHR for MS patients |
| Van Mens et al (2019) | Netherlands | Systematic review of reviews to provide a comprehensive review of determinants and outcomes of patient access to and control over their personal medical data, and the adoption of PP and PHRs | Primary, secondary or tertiary healthcare, any medical domain | Patients, informal caregivers, or healthcare professionals | PP provide patients with direct access to information in EHRs of clinicians. Electronic PHRs provide patients with their own system to manage their personal health information | Clinical Adoption Framework (CAF) |
| Otte-Trojel et al (2016) | Netherlands  Majority of studies North America (85/109) and Europe (14/109) | Review literature on PP development problems and solutions. Five specific research questions: 1) What categories of problems related to PP development defined? 2) What causal factors identified by problem analysis & diagnosis? 3) What solutions proposed to ameliorate causal factors? 4) Which proposed solutions have been implemented and in which organisational contexts? 5) Have implemented solutions been evaluated & what learning has been generated? | No specified care setting | No specified demographic or condition | A PP is a secure website through which patients can access personal health information and make use of communication, self-management and administrative functionalities. Included EHR-tethered portals and universal PHRs (as long as latter were clinically integrated, ie receiving information that originated in one or more EHRs) | Problem solving cycle (adapted from model of Van Aken et al 2012) |
| Jilka et al (2015) | UK  3 studies = USA, 1 = USA + UK, 1 = USA + Neths, 3 = mixed, 2 = unclassified | Review of reviews to evaluate critically the evidence regarding patient accessible EHRs (PAEHRs). Synthesise research to provide quantitative insight into impact of PAEHRs across range of outcome measures in different clinical populations, and to investigate differences between patients and HCPs | Mixed care setting - primary and secondary | Adult patients with chronic disease (eg diabetes and hypertension), HCPs | PAEHRS defined as patient accessible information held by physician and/or health care system and accessed by electronic means | Scoring system developed to weight impact of outcome measure quantitatively |
| Sakaguchi-Tang et al (2017) | USA  Geographical location of included studies not specified | Assess existing research landscape related to PP and ePHR use and experience among older adults & to understand the benefits & barriers to use & adoption. Questions: 1) What barriers & facilitators to older adults' use & adoption of PP and ePHRs have been described and what is the evidence for these factors? 2) How do older adults describe their experience of using PP and ePHRs? 3) What design recommendations have been proposed to overcome barriers & enhance facilitators? | No specified care area | Older adults age 60 and above | PP are systems for health information management that are tethered to a patient's EHR. Include features such as ability to schedule appointments, view test results, request prescription renewals and send messages to healthcare providers. ePHRs are not connected to EHRs and the individual is responsible for entering own health information. Include features such as health tracking or medication lists and ability to share health information with others. | No specific framework - focus on barriers, facilitators & experiences |
| Kneale & Demiris (2017) | USA  Majority of studies conducted in USA (n=7, 70%), Canada (n=2, 20%) and New Zealand (n=1, 10%) | To understand older adult perspectives on PHRs and to identify their self-reported needs by systematically examining publications that included older adult participants In PHR evaluations. Review explores characteristics older adult study participants and describes their views on the systems | No specified care area | Participants aged 60 and above | PHR is a system that allows consumers to access, manage and share personal health information over the internet. | No specific framework - focus on characteristics of older adult users, evaluation outcome measures & results, facilitators & barriers for PHR use |

**Multimedia appendix 1: Characteristics of included reviews (part 2)**

| **Reference** | **Databases searched** | **Inclusion & exclusion criteria** | **Data extraction method** | **Quality assessment method** | **Method of synthesis** | **Number of included studies** | **Summary findings** |
| --- | --- | --- | --- | --- | --- | --- | --- |
| D’Costa et al (2020) | Medline (Ovid), CINAHL (Ebsco), Scopus, reference lists of included studies scrutinised. Searches run February 2018 | Inclusion: adult hospitalised patients in acute setting, English, date range=1997 to February 2018; ethical issues search did not specify patient group and ran from database inception date to February 2018; Exclusion: paediatric studies, disease-specific studies, focus on confidentiality and data sharing, studies on system design | Setting, nature of study, sample size, nature and contribution of participants, nature of analysis, summary of results | Risk of bias mentioned in sub-heading p4 but no details of quality assessment are presented | Critical interpretive synthesis, thematic analysis of ethics literature | 18 papers representing 16 studies | 4 main themes from analysis: 1) impact on care, 2) conflicts between patient & physician perspectives, 3) divergent views of doctor & patient roles, 4) cultural differences & societal risks |
| Grossman et al (2019) | Medline (Ovid), EMBASE (Ovid), CINAHL (Ovid), Cochrane Reviews, searched personal reference libraries, scrutinised reference lists of included studies and pertinent reviews, searched table of contents of pertinent scientific journals between 1 May 2018 and 1 December 2018 to identify recently published citations | Inclusion: original research, interventions occur in 1+ vulnerable patient population (as per the PROGRESS-Plus criteria), patient portals, RCTs & other clinical trials, time series designs, pre-post designs, post only designs with two or more comparators, any other design with one or more comparisons, intervention aimed at increasing portal use or predictors of portal use or reducing disparities in portal use, outcomes (portal use, predictors, disparities), date range=inception of indexing of database to 31 August 2018, English, published or in press;  Exclusion: case series, case reports, perspectives, editorials, letters to editor, abstracts, reviews, in review or submission, white/grey literature, other consumer health IT (telehealth, eVisits, mHealth apps, paper-based interventions), interventions not intended to impact portal use | Study objective, setting, population, design, eligibility criteria, intervention category, findings | AHRQ Methods Guide for Effectiveness and Comparative Effectiveness Reviews | Descriptive analysis of study characteristics in Excel (with computed or estimated values if necessary), intensity of intervention assessed as per Cochrane Handbook for Systematic Reviews of Interventions, interventions categorised according to System Engineering Initiative for Patient Safety (SEIPS) model (ie which components addressed), meta-analysis and grade strength of evidence not possible due to paucity of literature and lack of directly comparable outcomes | 18 | Most interventions (72%) addressed only one component (the person) of SEIPS, no study intervened on more than two components; more studies assessed intervention impact on portal use than predictors of use, only 1 assessed impact on disparities in use. Participant characteristics not systematically reported. Best evidence for increasing portal use in vulnerable populations is technical training and assistance programmes, while other interventions are not sufficiently studied to draw conclusions |
| Kelly et al (2018) | PubMed, Web of Science, Cochrane, CINAHLPlus, Scopus, reference lists of included studies scrutinised | Inclusion: Date range=1 January 2006 to 8 August 2017, English; Exclusion: Studies of PP in emergency department or ambulatory setting and/or future study protocols, studies describing general inpatient technology or evaluating portals not tethered to inpatient EHR clinical data, qualitative studies describing development of portal prototypes and/or portal redesign | Study objective, design, setting, sample, data collection instruments, outcomes, and description of results | Downs and Black checklist for assessing methodological quality of randomised and non-randomised healthcare interventions | Analysed and described according to 3 themes of study objective | 17 | Content & design features recommended: 1) timely, personalised clinical & educational information in lay terms, 2) care trajectory (care plan & patient schedule), 3) way to recognise & communicate with healthcare team portal use |
| Dendere et al (2019) | PubMED, CINAHL, Embase; plus reference lists | Date range: 2005-2017 (plus periodic updates to Aug 2018); English; PP tethered to hospital EMR; hospital inpatient setting | Used 3 categories to classify data - inputs, processes and outputs; inputs = material (eg hardware and software) and non-material (eg leadership) components that facilitate or impair the establishment or use of PP; processes = interactions of users with PP; outputs = results of implementation or use of PP | A Measurement Tool to Assess Systematic Reviews (AMSTAR - 2), Quality Assessment Tool for Studies with Diverse Designs (QATSDD - empirical studies); assigned overall score on 5-pt scale (very low to very high) | 14 themes identified under 3 categories; Inputs - portal design, usability, barriers, facilitators, user training and organisational factors; Processes - adoption, use, information, and communication; Outputs - patient engagement, user perceptions, health outcomes, benefits; Valence ratings for overall findings for PP as positive, negative or mixed | 58; includes 12 reviews | Majority of studies reported to show mixed valence for PP, but more positive than negative; Barriers: poor portal design, patients' privacy concerns, lack of encouragement for adoption from providers, sub-optimal training; Facilitators: access to information, patient-provider communication |
| Aslani et al (2020) | PubMed, Scopus, Web of Science, IEEE, Embase, Proquest; general search in Google using key words; authors contacted for relevant studies | Inclusion: Date range: database inception to February 2019; articles and conference papers; articles about PHR attributes (contents and/or capabilities); articles on web-based PHRs for patients with MS; Exclusion: Educational reports, books and e-boosk, standards and industry papers; articles with no full-text available; articles not about PHR attributes; articles about non web-based PHR platforms | Data extraction form developed based on research objectives: general information (title, author, date, place, etc), objective & methods (objective, research type, data analysis, etc), main findings (attributes, must include contents and/or capabilities) | No quality assessment | Combined narrative and content analysis: information classified into categories and sub-categories | 39: developmental studies = 44% (17/39), descriptive studies = 31% (12/39), review studies = 23% (9/39) | Need to consider appropriate minimum data set (MDS) for PHR. Important capabilities include: data exchange & sharing, interoperability, accessibility, security & privacy, functionality, usability, architecture or implementation platform, data types |
| Van Mens et al (2019) | MEDLINE (including: Epub Ahead of Print, In-Process & Other Non-Indexed Citations; Medline R Daily; Medline R), EMBASE and PsycINFO - all via OVID; search limited to most recent publications until saturation was achieved regarding the summary measures | Inclusion: systematic literature reviews of studies with patients, informal caregivers or healthcare professionals in primary, secondary or tertiary healthcare, in any medical domain; interventions providing patient access to or control over their own medical records, or adopting or using PP and PHRs; primary outcomes of review focus on determinants (facilitators & barriers) or outcomes of patient access to or control over their own medical data; English; Date range= 2015 to 19 February 2018; Exclusion: articles with no abstract; publications earlier than saturation point for the summary measures (see method of analysis/synthesis) | Data extracted into spreadsheet. Information about the reviews: author, year, theoretical framework and critical appraisal method. Quotes were extracted from each article about determinants and outcomes of patient access to records, PP and PHRs. Any categorisation of the quotes (including any theoretical framework used in the original papers) also extracted. | AMSTAR2 checklist to assess risk of bias of the reviews, number of critical and non-critical flaws counted; no risk of bias on outcome level because should have been conducted in original reviews | Extracted quotes were mapped one determinant and one outcome category of CAF. Number of unique combinations of determinant & outcome categories calculated. Calculated how many reviews reported on each dimension and category and how many reviews reported how many relationships between different levels, dimensions and categories | 19 | Only 3 reviews reported on relationship between macro and meso level, most reviews reported on relationships between meso level and sublevels of micro level; all CAF dimensions were addressed by at least 2 reviews; all reviews addressed 'People' and 'Use' dimensions; there was less focus on higher level of standards, policy, funding & society influencing adoption; all reviews had several critical flaws |
| Otte-Trojel et al (2016) | PubMed, Science Direct, LISTA. Search conducted in January 2015 | Inclusion: peer-reviewed articles; English; date range=2005-2015; articles must concern PP that give patients access to PHRs, must address a problem encountered in PP development and/or must present a solution to a problem; qualitative & quantitative articles reporting primary research; population surveys and simulation studies focus on identifying problems or solutions; secondary research, including reviews, commentaries and conceptual articles; Exclusion: non-peer-reviewed articles; articles on PPs and universal PHRs not connected to EHRs | Information extracted relating to each of the 5 steps of the problem-solving cycle. Information extracted on each problem mentioned in article | States that quality of studies addressed, but no details provided | Abductive analysis approach (most simple & probable explanation of a given explanation). 1) Combined information about problem definition into problem categories. Compared categories to 2 relevant frameworks on PP development (Wakefield et al, 2010, Otte-Trojel et al, 2015). 2)Combined information about factors causing problems in each of the categories. 3) Ordered information about proposed solutions into themes describing a type of solution. 4) For implemented solutions, gathered information about solution & organisation context. 5) For implemented solutions that had been evaluated, took note of evidence from evaluation | 109; 61=primary research, 27=population surveys & simulation studies, 21=secondary research (reviews, commentaries & conceptual) | Five main problem categories identified: 1) achieving patient engagement 2) health service provider engagement 3) appropriate data governance 4) security & interoperability 5) sustainable business model. 45 articles proposed solutions to problems, but only 18 were implemented and only a small number (5) of evaluated designs - secure messaging curricula in residency training (1), strategies to promote portals to patients (2), patient training and guidance (2). Solutions can ameliorate problems of achieving patient and provider engagement, but cannot present evidence-based suggestions for solving problems in PP development due to modest number & relatively weak evidence. Patient engagement (especially socio-economic factors and PP use) and provider engagement are a main focus in literature. Few articles on financial sustainability. Uneven nature of evidence hinders PP development, especially as problems likely to be interrelated. Majority of implemented solutions within single organisations or organised care delivery systems which limits relevance to other organisational contexts (ie more challenging within fragmented care delivery contexts) |
| Jilka et al (2015) | Web of Science databases, including core collection, MEDLINE and BIOSIS Citation Index. Reference lists of included reviews examined for additional articles | Inclusion: English; date range = January 2002 to November 2014; systematic reviews that assessed PAEHRs on a variety of quality & clinically relevant outcome measures in adult populations; articles with suitable research questions, description of methods supporting paper as a review, and reported narrative on impact of PAEHRs; Exclusion: non-peer-reviewed, non-empirical, non-electronic use of record access, papers focused on design of PP system | Study characteristics (Author, title, year, journal, years of studies included, number of studies, patient/clinician oriented, geography, primary aim, findings) and scoring based on number of times a review reported on any outcome measure and the number of times these outcome measures were found to have a positive impact. Outcomes = usefulness/effectiveness, information quality, self-efficacy (patient empowerment, patient involvement, communication), patient satisfaction, patient views, patient engagement, clinical outcome, workload, psychological, doctor views, cost of interventions, self-reported outcome/behaviour, security, privacy & confidentiality concerns | Some commentary on number of RCT studies included in the reviews | Scoring of frequency & positive impact for outcomes across 16 domains | 10, but 8 in final analysis; 1 excluded because of duplicate citations and 1 excluded because it contained 32 citations not referenced directly within the outcome measures described in the paper | Mixed outcomes in respect of patient safety, usefulness, satisfaction, and self-efficacy across patients & HCPs. Small number of studies report potential for lower hospital costs. Lack of data driven evidence about opinions, wants and needs of large clinical consumer groups. Current research targeted to certain clinical groups (eg chronic disease) which makes findings difficult to implement across large non-disease-specific population |
| Sakaguchi-Tang et al (2017) | PubMed, EMBASE, CINAHL, Complete, Compendex (includes ACM Digital Library & IEEE Xplore), Inspec. Plus general search in Google Scholar. | Inclusion: English; date range = January 2006 to November 2016; studies include participation of adults 60 years or older (as sole focus of study or part of larger study); focus on PP and ePHRs; articles discuss use, adoption or experience with PP and ePHRs or with features of those systems (eg securing messaging with providers or access to medical records); formative studies focused on information gathering for design (including user testing of systems or assessments to inform development of systems or test acceptability of particular systems); Exclusion: studies not focused on use, adoption or experience; studies focused on patient online communities or provider experience of PP and ePHRs; papers that recorded only log-in data and demographics; non-empirical studies (including commentaries, letters to editors, notes, books, reviews and conceptual papers) | Key points extracted from summaries and from the papers | Mini Statement on the Reporting of Evaluation studies in Health Informatics (STARE-HI) - "Interpret the data & an answer to the study question", "Description of the outcome measure or evaluation criteria". Added two additional guidelines - "Provides a description of system & its functionalities" and "Provides clear description of how results impact design recommendations" | Thematic analysis - key point groups into codes (created using inductive process). Groups of codes refined into themes informed by research questions | 17 (representing 15 separate projects) | Commonalities among all papers concerning barriers & facilitators to  use & adoption of PP or ePHRs by older adults. More common for papers to describe barriers to PP use. Very few concrete solutions offered. Two main barriers across studies: 1) privacy & security, 2) access & ability to use technology & the internet. Two main facilitators: 1) technical assistance, 2) family & provider advice or recommendation; Evidence lacked strength (due to small sample sizes, convenience samples & diversity of studies); very few papers focused on facilitators in general & those that did looked at initial use; formative vs initial use may account more for pattern of results in relation to barriers and facilitators than the technology itself |
| Kneale & Demiris (2017) | MEDLINE (via PubMed), CINAHL (via EBSCO), PsycINFO (via EBSCO), EMBASE (via OVID), search conducted on 1 July 2015 | Inclusion: articles describing primary data analysis of a consumer evaluation; articles that focus on study participants aged 60 and above separately from other adult populations (articles included if age categories of 60 and older included at least 50% of total participants; articles that evaluated a PHR according to review definition (a system that allows consumers to access, manage & share personal health information over the internet); date range = inception to July 2015; English; Exclusion: participants not exposed to a PHR; participants only exposed to PHR in a laboratory setting | Study design, patient population, study setting, PHR features, participant demographics, evaluation outcome measures and results, facilitators & barriers for PHR use among older adults. | 3 most important items from condensed version of the Statement on Reporting of Evaluation Studies in Health Informatics (STARE-HI). Added two additional criteria: provides a description of the PHR functionality under investigation and describes how participants used the PHR functionality under investigation. Ranked on a scale 0 (worst) to 5 (best). | Not described - focus on participants demographics, evaluation outcomes, barriers & facilitators | 10 (covering seven unique PHRs) | Many PHR evaluations do not include a diverse range of older adults, leading to gaps in understanding of user needs & possible contribution to disparities in adoption rates. Older adults perceive PHRs to be helpful to their care & are willing to overcome technical barriers to engage with systems. |
